# The Jewish Perspectives on Xenotransplantation

**DOI:** 10.5041/RMMJ.10511

**Published:** 2023-10-29

**Authors:** John D. Loike, Rabbi Moshe Krupka

**Affiliations:** 1 Co-Director of Bioethics, New York Medical College, Valhalla, New York, USA; 2 Professor of Biology and Bioethics, Touro University, New York, New York, USA; 3 Executive Vice President and University Ombudsman, Touro University, New York, New York, USA

**Keywords:** Bioethics, Jewish law, xenotransplantation

## Abstract

Xenotransplantation represents a viable solution to meet the great need to provide organ donors at a time when there are not enough human organ donors. A lot of clinical studies have focused on using genetically engineered pigs as the prime source for organ transplantation. However, several religions, such as Judaism and Islam, have restrictions on the use of pigs for food or in business. In this article, we review the Jewish perspectives on xenotransplantation. Overall, the preservation of human life trumps most of the potential religious concerns associated with xenotransplantation. However, there are religious nuances related to xenotransplantation that are highlighted here, and that must be addressed by rabbinical scholars.

## INTRODUCTION

Only about 50% out of 100,000 Americans waiting for transplants each year will find a human donor. Nearly 37 million American adults suffer from chronic kidney disease, with 786,000 of those diagnosed with end-stage renal disease (ESRD). Those with ESRD and 4.5 million adults in the US diagnosed with liver disease face a ticking clock as they await life-saving transplants.^1^ In addition to the human and medical need for these patients to receive organ transplants, the financial costs to maintain these patients are staggering. The American Society of Nephrology reports, for example, that Medicare annually spends $35 billion treating kidney failure and $114 billion managing kidney diseases.

Xenotransplantation, or cross-species organ transplantation, is currently being developed as a means to alleviate this tragic medical problem. Xenotransplantation involves the transplantation or infusion into a human recipient of either organs, tissues, or live cells from a non-human animal source such as a pig.

In the last year, there has been a great deal of progress in applying xenotransplantation to humans in need of an organ. The first-ever landmark gene-edited pig heart transplant to a human occurred in January 2022. The patient, David Bennett, was deemed ineligible for human heart transplantation and mechanical circulatory support, based on a history of non-compliance. He received a gene-edited pig heart and survived for 50 days. After his death, the research team learned that the transplanted organ was infected with a pig herpesvirus that had not been detected by pre-transplantation tests.^2^

A few months after the heart transplant, scientists independently reported^3^ transplanting the first pig kidneys into three people who had been declared legally dead because they lacked central brain function. These studies showed that the transplanted kidneys in these subjects produced urine and were not rejected by the human immune system, even two to three days after the procedure. In June and July of 2022, surgeons performed two more pig heart transplants on brain-dead people, and we are awaiting the outcomes of these procedures.

While pig-to-human transplants are relatively new, hundreds of pig-to-baboon transplants have been done over the past 50 years. The organs transplanted were mostly hearts, kidneys, and insulin-producing islet cells, and many of these baboons lived for several years.^4^,^5^ In the 1990s and earlier, pigs were chosen as potential organ donors for xenotransplantation because they are available in large numbers, are easy to breed, and are more likely to receive public acceptance over non-human primates. Pigs are well-suited candidates as animal organ donors because their organs are similar in size and anatomy to human organs.

Animal organs also have some clinical advantages over human equivalents because there is no need for the patient and a surgical team to be available at a moment’s notice when a genetically compatible human donor dies. In addition, it is much easier to screen animals, such as pigs, to determine their health as organ donors.

One of the significant obstacles in pig-to-human xenotransplantation is the risk of organ rejection by the recipient. However, it was discovered that mutating a pig gene encoding a protein that triggers human rejection (α-Gal) allows organs transplanted from these modified animals to survive much longer in non-human primates. Moreover, new gene-editing technologies such as CRISPR will reduce some of the risks associated with xenotransplantation. The pig whose heart was transplanted into David Bennett was engineered by Revivicor and had 10 genetic changes, including four human genes that suppress the immune response and two genes that prevent blood from coagulating in response to inflammation. Other companies are engineering pigs with altered genes to prevent the development of preformed and human neo-antibodies against non-human leukocyte antigens, the presence of which can lead to organ rejection and decreased long-term graft survival.^6^

Not all potential animal organs will require extensive genetic engineering. For example, pig pancreata lacking the immunogenic sugar (α-Gal) and containing two extra genes that dampen the human immune response were used in pig-to-baboon transplants. These transplanted pancreatic islets cured diabetes in five baboons, who lived for nearly two years without insulin or even constant immunosuppressive drugs.^7^

Another health concern about using pigs as organ donors is that pigs contain porcine endogenous retroviruses (PERVs). These viral elements embedded in the pig genome are harmless to pigs but might be harmful when transmitted to people receiving the transplant leading to a disruption of normal gene function or oncogenesis.^8^ Efforts to eliminate these PERVs have proven successful by using CRISPR gene-editing technologies to scramble all known PERVs in the pig genome.^9^

The costs of xenotransplantation must also be considered. Genetically engineered pig organs are not inexpensive because these engineered animals must be grown in carefully controlled and quarantined settings. The caretakers for the pigs must wear what are essentially spacesuits to ensure the animals do not get infections, and they must monitor the pigs frequently to ensure they are not carrying diseases.

There are additional monitoring costs in xenotransplantation. The patients will not only be monitored to ensure that their immune system does not reject the animal organ but also to ensure these patients do not contract any sort of post-transplantation infection. In addition, patients will have to be monitored to determine whether they acquire novel zoonotic diseases from the pig that could potentially spread to others, creating a new viral pandemic.

Currently, the cost of a heart transplant in the United States is around $1.66 million, according to the most recent estimates.^10^ Some scientists claim that the price of a pig heart transplanted into a baboon is only $500,000.^10^ Unlike pig transplants, transplanting a human organ is more expensive because of the costs associated with keeping a deceased donor’s organs viable during transport and the need to fly out transplant surgeons and other experts at a moment’s notice.

The reduced expense of xenotransplants is also due to the fact that potential recipients no longer have to wait in an intensive care unit for months or be on dialysis for years as they wait for a human organ to come available. Most importantly is that the cost in terms of saving human lives is immeasurable.

## ETHICAL ISSUES ASSOCIATED WITH XENOTRANSPLANTATION—A JEWISH PERSPECTIVE

### Animal Rights

Xenotransplantation has re-sparked a debate over using pigs for human transplants, which many animal rights groups oppose because it is “unethical, dangerous, and a tremendous waste of resources.”^11^ In addition, these animal rights groups, like PETA, claim that pigs “aren’t tool-sheds but complex, intelligent beings.”^12^

Jewish law specifically prohibits animal suffering. Causing any unnecessary pain to animals is prohibited as stated in the Bible: “You shall not see your brother’s donkey or his ox fallen [under its load] on the road, and ignore them.”^13^ In fact, Judaism mandates that we help unload an overburdened pack animal as quickly as possible. Moreover, the Bible demands that all domesticated animals must be fed before their owner sits down for a meal. In addition, animals belonging to Jews must not do any work on the Sabbath—a commandment clearly stated in the Holy Decalogue. Preventing animal suffering is so critical that one may desecrate the Sabbath to relieve the suffering.

However, the ethical guideline of respecting human dignity and preserving human life dictates that animals should be sacrificed for food or medical applications to preserve or save the life of a human being.

Human beings enjoy a unique and higher dignity than other animals because God created human beings “in the image of God.” The elevated value of human life is reflected in the moral teachings of the Talmud, which states: “Adam [from whom all humanity descended] was created singly, to teach us that whoever destroys a single life is considered by Scripture to have destroyed the whole world and whoever saves a single life is considered by Scripture to have saved the whole world.”^14^ Using engineered pigs as kidney donors would fall under the precept “whoever saves a life saves the world” and will be accepted by the leading Jewish legal scholars. In fact, using porcine aortic valves for transplantation is universally accepted and permitted by all leading rabbinical scholars.^15^ Thus, the preservation of human life usually trumps all other ethical concerns. In fact, as early as the 1500s, rabbinical scholars ruled that animals can be used in medical research. For example, animals can be used to test the health benefits and side effects of a newly developed drug.^16^

### Use of Non-Kosher Animals in Medicine

Like Islam, Judaism has strict rules on using pigs as food or as products in a business venture. This law is based on the Biblical verse:

And the swine—although it has true hooves, with the hooves cleft through, it does not chew the cud: it is impure for you. You shall not eat of their flesh or touch their carcasses; they are impure for Me.^17^

Jewish law permits a Jew to own non-kosher animals but prohibits consuming meat or milk from non-kosher animals, such as pigs. Moreover, Jews cannot engage in business ventures using non-kosher foods or non-kosher animals that are usually raised for human consumption.^18^ The Talmud^19^ and Code of Jewish Law^20^ specifically state that one is not permitted to breed or raise pigs in any place (as a business), whatsoever. Interestingly, Judaism believes that all of God’s creations exhibit some degree of holiness. Thus, non-kosher animal products were used to beautify the Holy Jerusalem Temple.

As stated above, according to Jewish law, any commandment in the Bible (except idolatry, murder, and forbidden sexual relationships) should be violated to save a person’s life; This means that there is no violation in using pig organs to save a human life.^21^ In fact, for decades, rabbis allowed the use by diabetics of insulin made from pigs or the use of porcine heart valves to be transplanted into a person.^11^

## CORE VALUES OF HUMAN–ANIMAL EXPERIMENTATION

Judaism teaches that human beings must always consider whether a new technology will better the world or elicit destruction. The Bible emphasized the directive to humankind to partner with God in completing the creation process and protecting the earth, all life forms, and the environment. Jewish laws that leave the land fallow such as the Sabbatical year and the Jubilee,^22^ are partly designed and formulated to preserve and rest the land, to respect plant life, and to ecologically benefit the land and water. Partnering with the Divine in protecting his creations reflects the Jewish notion of stewardship as a moral category. The secular issue of whether human beings should “play God” is not an issue in Judaism. In fact, partnering in creation with God is a moral/religious directive that speaks of the responsibility rather than of unlimited privilege as emphasized in Genesis.^23^

The partnership agreement between humankind and God is not without limits. Humankind does not possess the *carte blanche* right to engage in any scientific experiment that we desire. This is one lesson that Judaism learned from the story of the Tower of Babel: humankind should never let infatuation of technology override moral values.

The Kabballah states that “God created the first human, He took him and led him around all the trees of the Garden of Eden, and said to him, ‘Behold My works, how beautiful and commendable they are! All that I have created; I created for your sake.’”^24^ The lesson derived from this legend is explicit: we must pay heed that we do not corrupt and destroy the universe; for there is no one to repair it.

How does Judaism determine whether a technology should be pursued or the direction of its development? Here rabbinical authorities will evoke a basic principle of logical reasoning in rendering legal decisions. Various Talmudic texts^25^–^27^ emphasize that logical reasoning is essential in establishing legal decisions when there are no explicit prohibitions in Jewish law.

One issue with xenotransplantation is the need to raise millions of donor pigs to serve as donors for the millions of people who need organ transplants. Thus, Judaism demands that rabbinical scholars consider the impact of technology on the environment.^28^ Rabbis will consider the impact on the environment of raising engineered pigs, to ensure that everything is done to reduce the negative effects.

Another Jewish legal question relates to whether people should and can volunteer for clinical xenotransplantation studies. There are at least two situations where Jewish law discusses this situation. The first is whether one is permitted to volunteer as a heart, kidney, or pancreas recipient before procedures are FDA-approved. The second is whether one is permitted to donate one’s body, after death, to better understand the methods of organ xenotransplantation.

In the first case, Judaism encourages but does not demand volunteering for clinical studies. Each person has the autonomous right to choose. If a person is critically ill, there is more religious justification to engage in a clinical trial. Some rabbis believe that any chance to save a life, however remote, must be pursued at all costs.^29^ Some rabbinical authorities believe that “if the gamble is great and the potential gain small, such as in phase I or II trials, participation is not mandated and may even be forbidden”—although participation in a phase III trial would be permissible. However, if a person refuses to receive an animal transplant on any potential religious grounds, they should not be given less priority on waiting lists for human organ donors. Clearly, these questions will necessitate the direct guidance and halachic jurisprudence of recognized halachic experts.

The issue of donating one’s own body for medical research as was done to study kidney xenotransplantation is not easily resolved in Judaism. The main problem is that Judaism adheres to the belief in the inviolability of the human body. The Talmud^30^ asserts that the biblical imperative of speedy burial^31^ is based upon the prohibition of disgracing a corpse and includes any form of injury or postmortem dissection to the body. However, the Jewish prohibition against the performance of autopsies does have an exception—if the autopsy may *directly* contribute to saving the life of another patient who is currently awaiting treatment or if there is a pandemic where doing an autopsy could save many lives. Thus, rabbinical scholars will need to ascertain whether or not the use of cadavers to better develop xenotransplantation protocols could be classified as an exception to the Jewish law forbidding the desecration of a corpse.

## OTHER UNRESOLVED JEWISH ETHICAL ISSUES

Rabbis are always concerned about the psychological state of the patient asking for a legal decision and how potential stigma issues may influence the psychological state of a patient. While, for example, pregnancy terminations are only allowed if the pregnant mother is in medical danger, psychological trauma of the pregnant woman can play a critical role in allowing fetal termination. Thus, in the case of xenotransplantation, most rabbis will adjudicate these situations on a case-by-case basis to ascertain whether a person should undergo the procedure. In many previous situations, the recommendations are that several physicians and rabbis are consulted before xenotransplantation is FDA-approved.

It is important to mention one other ethical challenge regarding xenotransplantations: its effect on human organ trafficking.^32^ Trafficking people for organ removal is a form of trafficking in which individuals are exploited for their organs, by coercion, deception, or abuse of a position of vulnerability. The non-governmental organization (NGO) Global Financial Integrity (GFI) estimates that the annual value of organ trafficking ranges from $840 million to upwards of $1.7 billion globally, and that about 12,000 illegal transplants occur each year, around 8,000 of which are for kidneys. It is not difficult to predict that if xenotransplantation becomes an effective therapy to save human life, the number of illegal transplants would decrease dramatically. Rabbinical scholars would thus be further encouraged to permit xenotransplantation to decrease global organ trafficking.

There are other ethical issues that will require further rabbinical analysis and resolution. For example:

- Autonomy—the right versus duty to engage in medical interventions that have not yet been approved by the FDA or are in clinical trials.
- Defining and assessing human and animal pain and the degree of pain and patient suffering that results from transplants.
- Determining how much pain and suffering a patient must bear when agreeing to medical interventions or a clinical trial such as in the case of xenotransplantation.
- Defining medical success (e.g. increased longevity and quality of life) following an organ transplant.
- Allocation of resources to the served and unserved populations that require heart transplants.

## CONCLUSIONS

Clearly, Jewish law greatly values saving a life, and xenotransplantation offers a therapeutic procedure that will save many lives. One important outcome from all the future clinical studies is assessing the viability of the patient receiving the transplant. The hope among most transplantation surgeons is that these treated patients will live for years. In Jewish law, rabbis will always take into consideration whether the patient will live longer with his own organ or with the newly transplanted pig organ. If our hope for xenotransplantation is realized, then longevity of the transplant will not be an issue.

## Abbreviations

ESRD: end-stage renal disease
PERVs: porcine endogenous retroviruses
